# Efficacy and safety of Etanercept therapy in juvenile idiopathic arthritis

**DOI:** 10.1186/1546-0096-9-S1-P66

**Published:** 2011-09-14

**Authors:** Arina Lazareva, Valda Stanevicha, Jelizaveta Semjonova, Ruta Shantere, Inita Buliņa

**Affiliations:** 1 Riga Stradins University, Riga, Latvia; 2 Children University hospital, Riga, Latvia; 3 P. Stradins University hospital, Riga, Latvia

## Background

Juvenile idiopathic arthritis (JIA) is the main cause of chronic arthritis in children who may continue to have active disease in adulthood. In Europe JIA patients resistant to traditional therapy started to receive treatment with biological drug Etanercept (ETN) in 2000. In Latvia ETN therapy was started in 2004.

## Aim

To evaluate the efficacy and safety of ETN therapy in the traditional treatment resistant JIA patients.

## Methods

The study included data from out-patient medical record on 69 JIA patients (45 children, 24 adults) who received ETN therapy during 2004 till 2010. Treatment efficacy was assessed using the ACR (American College of Rheumatology) Pedi 30, 50 and 70 response. Therapy was evaluated after 3, 6, 12 and 24 months (mo) of ETN treatment. Safety was assessed by recording ETN adverse events (AE). Statistical data analysis was made using Friedman, Kolmogorov-Smirnov and Pearson's chi-square tests.

## Results

Enrolled JIA patients were 4 to 23 years old. 65 patients (94.2%) had the sero-negative polyarthritis, 2 (2.9%) had seropositive polyarthritis and 2 (2.9%) systemic onset JIA. The mean disease duration before ETN therapy was 4.34 years (SD 2.50) and average ETN therapy duration was 2 years for children and 2,88 years for adults. After 3 mo of ETN treatment 70% of patients reached ACR Pedi 30 response, ACR Pedi 50 and ACR Pedi 70 were reached in 20% and 10%, respectively. Effficacy continued to improve and after 12 mo of ETN therapy ACR Pedi 30 and 50 responses were achieved in 100% of patients and ACR Pedi 70 response was reached in 85.7%. Significant ACR Pedi improvement observed at the end of the first year of ETN therapy, compared to 3 and 6 mo of treatment (p = 0.002).

Etanercept side effects were detected in 12 of 69 (17.4%) patients of whom 4.35% were children. They had mild AE - upper respiratory tract infections and uncomplicated herpes zoster infection. In adults AE observed in 13.05% cases – mild upper respiratory tract infections, headache, chronic uveitis relapse, infectious mononucleosis syndrome, and one opportunistic infection – tuberculosis.

## Conclusion

1. Etanercept is an effective therapy for traditional treatment resistant JIA patients. 2. Etanercept treatment efficacy gradually increased between 3 and 6 mo of therapy, reaching a maximum effect at the end of the first year of treatment. 3. Treatment with Etanercept in childern was safe, but in adults one JIA patient was diagnosed with opportunistic infection - tuberculosis.

